# Supplementary material for: Destabilisation of the Subpolar North Atlantic prior to the Little Ice Age
Source: Nat Commun. 2022 Aug 25;13:5008. doi: 10.1038/s41467-022-32653-x (PMC9411610; doi:10.1038/s41467-022-32653-x)
Supplement: Supplementary file 1 — Supplementary Information [file 41467_2022_32653_MOESM1_ESM.pdf]

Destabilisation of the Subpolar North Atlantic prior to the Little Ice Age

Supplementary Information

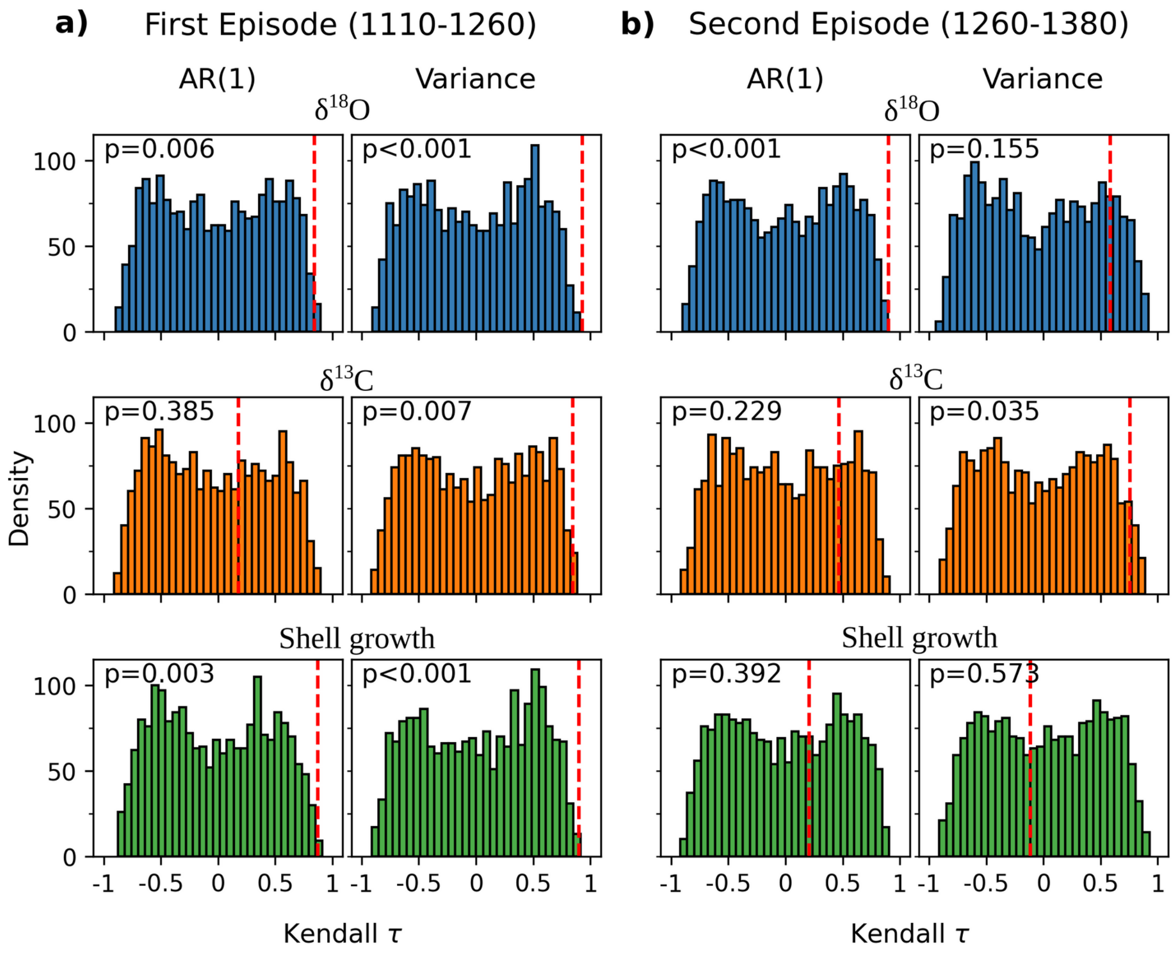

Supplementary Figure 1. Distribution of Kendall τ values expected from ensembles of 3,000 realisations of a null model obtained by sampling with replacement from the residuals of each original time-series during **a)** the first episode, **b)** the intermediate period, and **c)** the second episode. Significance tests for $\delta^{18}O_{shell}$ are represented in the upper panels, $\delta^{13}C_{shell}$ in the middle panels and Growth Index on the lower panels. AR(1) and variance were computed along a 70-year sliding window after detrending with a 40 years bandwidth each surrogate series. The red dotted vertical line indicates the Kendall τ value measured in each original record.

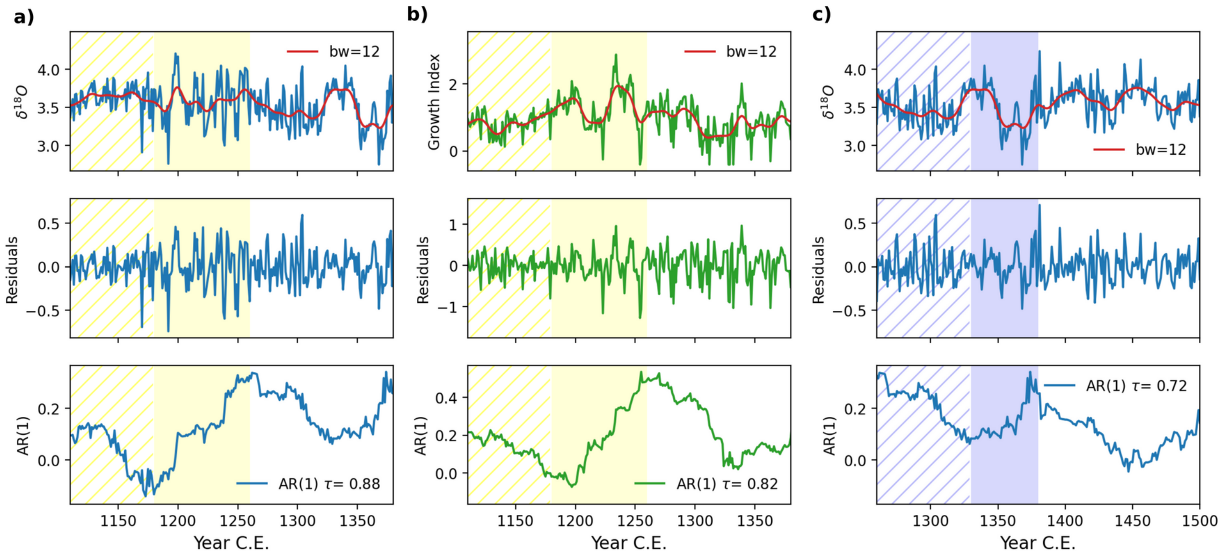

Supplementary Figure 2. Trends in AR(1) after detrending using a short bandwidth. Records are detrended using a bandwidth of 12 years to remove the irregular oscillations that characterise each episode. The annual values and the estimated trend (red line) are represented on the upper row. The middle row shows the residuals after detrending and the bottom row the AR(1) values obtained using a window length of 70 years. **a)** $\delta^{18}O$ and **b)** Growth Index records during the first episode. c**)** $\delta^{18}O_{shell}$ during the second episode. Declining resilience is detected over the yellow and blue shaded intervals corresponding to the first and second episodes respectively. The hatched region comprises the initial window interval for each episode.

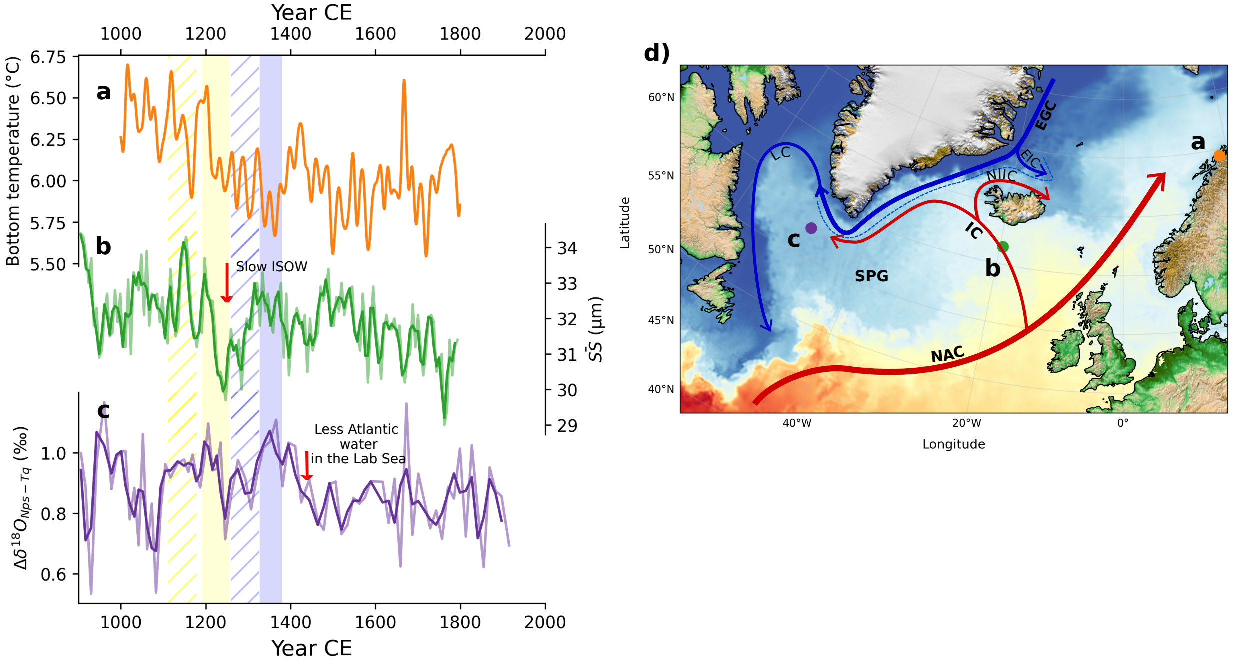

Supplementary Figure 3. a) Reconstructed sea bottom temperature from benthic foraminiferal $\delta^{18}O$ at Malangen Fjord as a proxy for the influence of the Atlantic warm inflow[1]. **b)** Sortable silt mean grain size ($\overline{SS}$) record from South Iceland as a proxy for near-bottom flow speed of the Iceland-Scotland Overflow Water (ISOW), which is influenced by the strength of the warm Atlantic inflow[2]. **c)** Difference in $\delta^{18}O$ measured on the foraminiferal species *N*. *pachyderma* and *T*. *quinqueloba* ($\Delta\delta^{18}O_{Nps\text{-}Tq}$) in the Labrador Sea as a proxy for the influence of warm Atlantic waters influenced by the SPG strength[3]. **d)** Location of the proxy records shown on the left figure (a-c) and regional ocean circulation. The colour map represents April sea surface temperatures during 2016, obtained from the ESA Climate Change Initiative data[4]. The land image was obtained from the NOAA National Oceanographic Data Center[5].

Supplementary Table 1. Information regarding the different proxy records selected in this study and shown in Figure 4 and Supplementary Figure 3.

Site Name	Reference	Proxy	Climatic parameter	Age model	Dating Uncertainty	Resolution over 1000-1450 CE
North Icelandic Shelf	Massé et al. (2008)[6]	IP_{25} of sea-ice diatoms	Sea ice	Tephrochronology & AMS ^{14}C	±1 to ±50 years	2 to 12 years Avg: 4.5 years
Norwegian margin	Sejrup et al. (2011)[7]	$\delta^{18}O$ of the planktonic foraminifera *Neogloboquadrina pachyderma*	Summer SST	^{210}Pb & AMS ^{14}C & tephrochronology	±20 years	2 to 26 years Avg: 9.5 years
Norwegian margin	Andersson et al. (2003)[8]	$\delta^{18}O$ of the planktonic foraminifera *Neogloboquadrina pachyderma*	Summer SST	^{210}Pb & AMS ^{14}C	±25 to ±60 years	Max: 34 years Avg: 9.1 years
Norwegian margin	Berner et al. (2011)[9]	Diatom assemblages	August SST	^{210}Pb & AMS ^{14}C	±30 to ±100 years	1 to 11 years Avg: 6.9 years
South Iceland	Moffa-Sánchez et al. (2014)[10]	Paired Mg/Ca–$\delta^{18}O$ from the planktonic foraminifera *Globorotalia inflata*	Annual SST	AMS ^{14}C	±100 to ±169 years	Avg: 6.2 years
North Icelandic Shelf	Jiang et al. (2015)[11]	Diatom assemblages	Summer SST	Tephrochronology & AMS ^{14}C	±1 to ±50 years	5 to 17 years Avg: 10 years
Ellesmere Island, Canadian Arctic	Lapointe et al. (2020)[12]	Titanium in laminated sediments	Atlantic Multidecadal Variability	Varve counts & Paleomagnetic inclination &OSL dating & 137CS &^{210}Pb	Precisely dated	Annual
Rockall Trough, Northeast Atlantic	Copard *et al.* (2012)[13]	εNd from deep-sea corals	Relative influence of different water masses	$^{230}Th/U$	±6 to ±120 years	2 to 179 years Avg: 44.5 years

Subpolar North Atlantic	Moffa-Sánchez & Hall (2017)[3]	Sortable silt mean grain size (10–63 µm fraction)	Near-bottom flow speed of Iceland Scotland Overflow Waters and subpolar gyre strength	^{210}Pb & AMS ^{14}C	±41 to ±71 years	Avg: 6.35 years
Norwegian margin	Tegzes et al. (2017)[14]	Sortable silt mean grain size (10–63 µm fraction)	Strength of the Norwegian Atlantic Slope Current	^{210}Pb & AMS ^{14}C	±128 years	1 to 32 years Avg: 6.7 years
Malangen Fjord	Hald et al. (2011)[1]	$\delta^{18}O$ of benthic foraminifera	Bottom water temperature related to the influence of the warm Atlantic inflow	^{210}Pb & AMS ^{14}C	±100 to ±140 years	Max: 9.62 years Avg: 3.5 years
South Iceland	Moffa-Sanchez et al. (2015)[2]	Sortable silt mean grain size (10–63 µm fraction)	Near-bottom flow speed of Iceland Scotland Overflow Waters	AMS ^{14}C	±100 to ±169 years.	Avg. 6.2 years.
Northeastern Labrador Sea	Moffa-Sánchez & Hall (2017)[3]	Difference in $\delta^{18}O$ measured on the foraminiferal species *N.* pachyderma and *T. quinqueloba*	Influence of warm Atlantic waters	^{210}Pb & AMS ^{14}C	±28 to ±101 years	9 to 19 years Avg: 12.3 years

Supplementary Note

Effect of the variable number of replicates and ontogenetic trends on the resilience trends

Before estimating changes in resilience, we reviewed the methods used to construct each proxy to assess their potential effects in autocorrelation and variance. The isotope series were obtained by averaging the number of replicate samples each year, which varies between one and six throughout both records[15,16]. In this case, we could expect intervals with a smaller sample size to have the largest variance. In both series, the number of replicates before 1170 CE is mostly one and increases to two between 1170 and 1240 CE[15,16]. If the sample size effect were the primary factor driving the variance trend, it would decrease over the first episode as the number of replicates increases; instead, a positive trend is observed.

The construction of shell-growth records involves more steps because shell-growth is influenced by age: young bivalves grow faster and produce wider and more variable annual increment bands compared with those produced during the mature years. Butler *et al.*[17] built two versions of the same record using different approaches to remove the age-related trend. The first method fits a negative exponential curve to the measurements of each shell after stabilising variance, whereas the second one follows the Regional Curve Standardisation (RCS) method developed in dendrochronology. Both versions exhibit similar trends in AR(1) and variance (Supplementary Fig. 4c,d), indicating that neither approach affects the trends in the indicators. However, the variance stabilisation methods do not entirely remove the ontogenetic trend. We assess the persistence of the age-related trend by computing the resilience metrics over the individual shell-growth records. Before computing the trends in the indicators, the age-related trend is removed by fitting a negative exponential curve after stabilising variance using a data-adaptive power-transformation method[18]. The results indicate that the ontogenetic trend in variance persists, resulting in significant negative trends in this indicator (Supplementary Fig. 5b). The earliest years are also slightly more autocorrelated, resulting in small negative trends in AR(1) (Supplementary Fig. 5a). In this scenario, the age-related trends might modify the trends in the indicators over the intervals where new shell records are introduced to the chronology. The first episode coincides with the successive introduction of five shells (Supplementary Fig. 4e), possibly altering the trend in the indicators. To investigate whether the trends respond to the introduction of new shells, we analysed the individual shell series around 1200 CE. The individual series share a common trend and similar values for both metrics

between 1200 and 1260, regardless of their age (Supplementary Fig. 6), suggesting that the ontogenetic trends do not drive the observed signal.

In addition to the age-related effects, the variance trends can also be affected by the variable number of replicates through time, expecting to observe increased variance over regions with the smallest sample size. To assess the probability of obtaining the observed signal due to the combination of both effects, we compared the observed trends to those obtained from three null models. Each null model comprises 1,000 surrogate series, and each series was created by introducing randomly chosen individual shell records at the same time as in the original chronology between 1100 and 1260. The probability of obtaining the observed trend under each scenario is measured as the proportion of surrogate series that yield higher or equal Kendall τ values as those obtained from a series built in the same way but preserving the original order in the shell records.

The first null model aims to test only the age-related effects without considering the effect of the variable number of replicates. In this model, the indicators are computed over the individual random records and annually-averaged to obtain each surrogate series. The results from this experiment indicate that it is possible to obtain positive trends in AR(1) and variance purely associated with age-related effects (Supplementary Fig. 7a). However, the proportion of series that yield larger values than the measured in the original records is less than 0.02 for both metrics.

The second experiment assesses the effect of the variable number of replicates through time on the indicators. In this case, each surrogate series is generated by averaging synthetic white-noise series of the same length, mean and variance as the shell records from the original chronology. This experiment demonstrates that the variable number of replicates significantly affects the variance trends (Supplementary Fig. 7b), resulting in negative trends because the number of replicates starts with one and increases progressively to six before 1260 (Supplementary Fig. 4e).

The third null model tests the effect of both factors combined. Each surrogate series is created by averaging the individual records using a biweight robust mean, as in the original records. The resilience indicators are then computed over the resulting series. In this experiment, the trends in AR(1) do not exhibit a positive bias, suggesting that autocorrelation is destroyed by averaging the series unless the individual records

contain the same signal. On the other hand, variance is slightly skewed towards negative trends (Supplementary Fig. 7c), indicating that the effect of the variable number of replicates outweighs the age-related effects. The proportion of surrogate series that yield higher values than the observed in the original record is less than 0.002 for each metric. The results from the null models indicate that it is improbable that the observed trend during the first episode resulted from age-related effects or due to the variable number of replicates, supporting the idea that the environment is the primary driver of the observed signal before 1260.

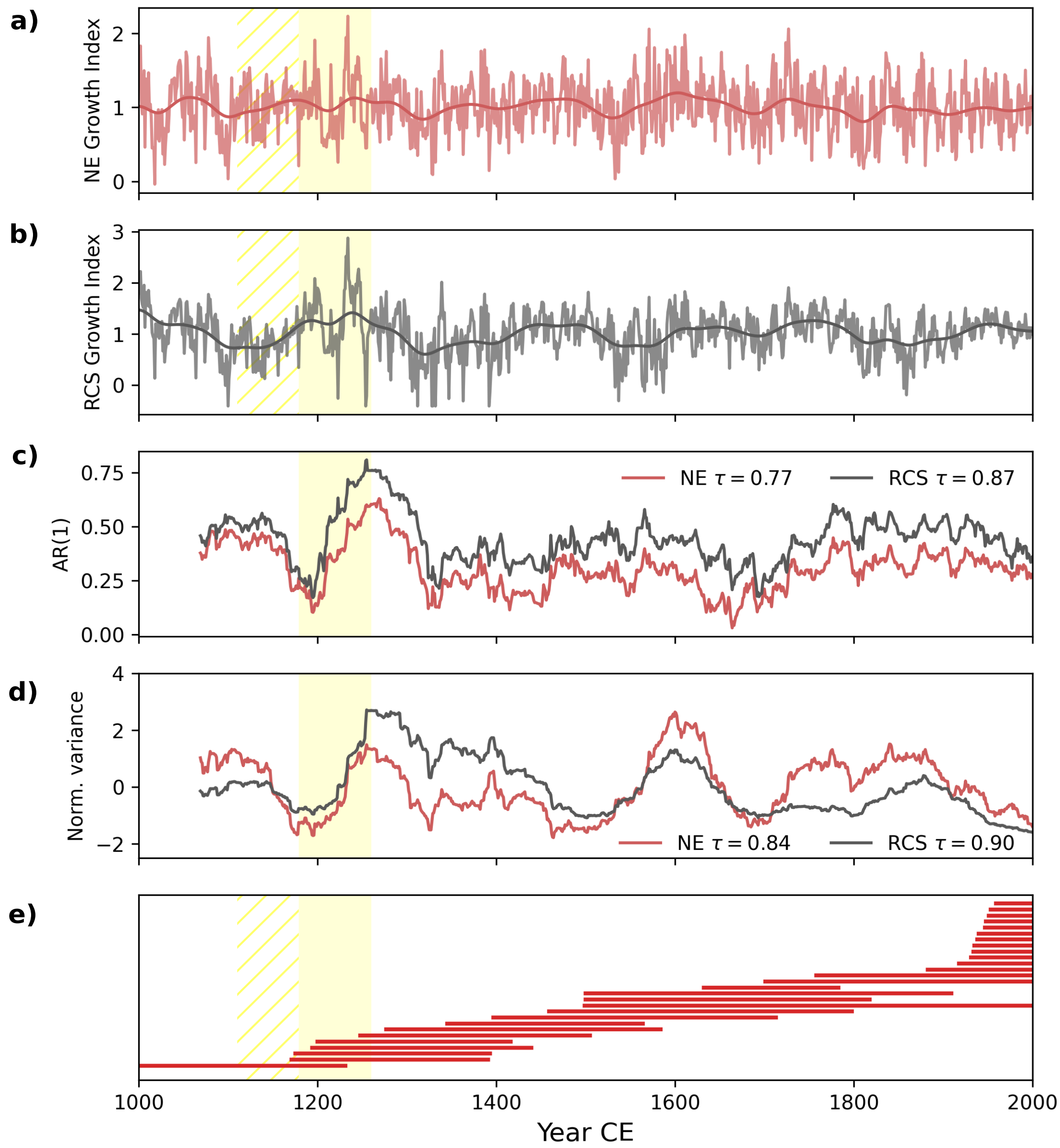

Supplementary Figure 4. Resilience indicators measured over two different versions of the shell-growth record. a) Negative exponential (NE) version. **b)** Regional Curve Standardisation (RCS) version. **c)** AR(1) and **d)** variance trends obtained using a window length of 70 years and a bandwidth of 40 years (see Methods). **e)** Lifespans of the shells used in the chronology. The first episode of loss of resilience is shaded in yellow. The hatched region comprises the initial sliding window interval.

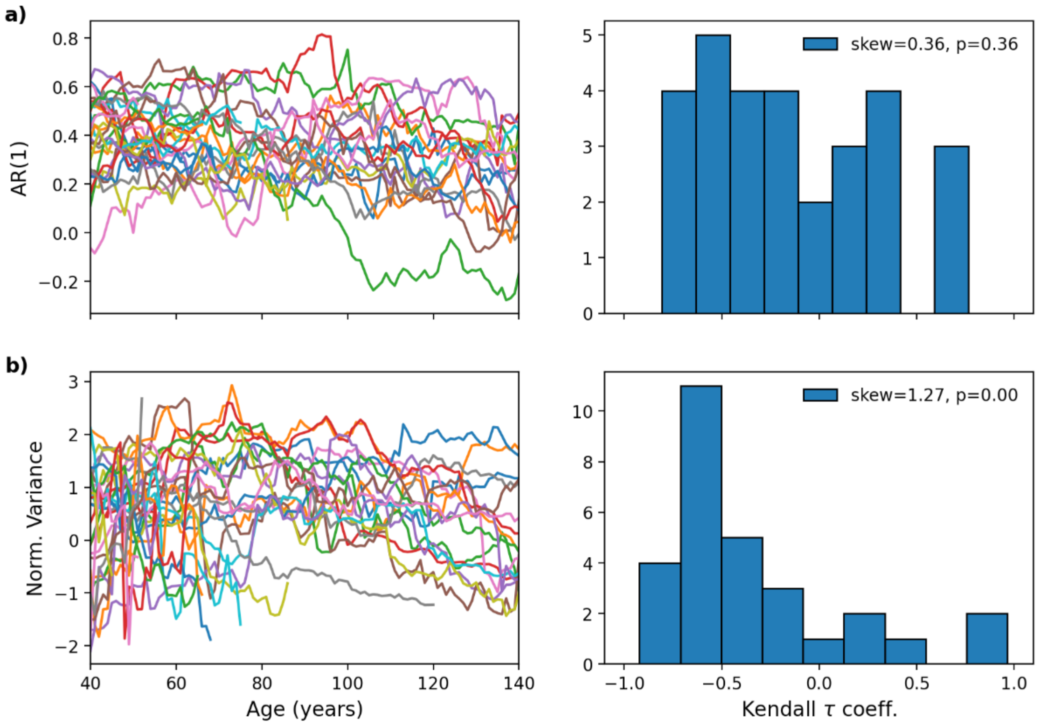

Supplementary Figure 5. Ontogenetic trends in the resilience indicators. a) AR(1) and **b)** variance values as a function of the bivalves age are shown on the left column. The right column shows the distribution of Kendall τ values for each indicator. AR(1) and variance were computed along a 40-year window after detrending with a 40 years bandwidth. The p-value represents the probability that the measured skewness is the same as that of a corresponding normal distribution.

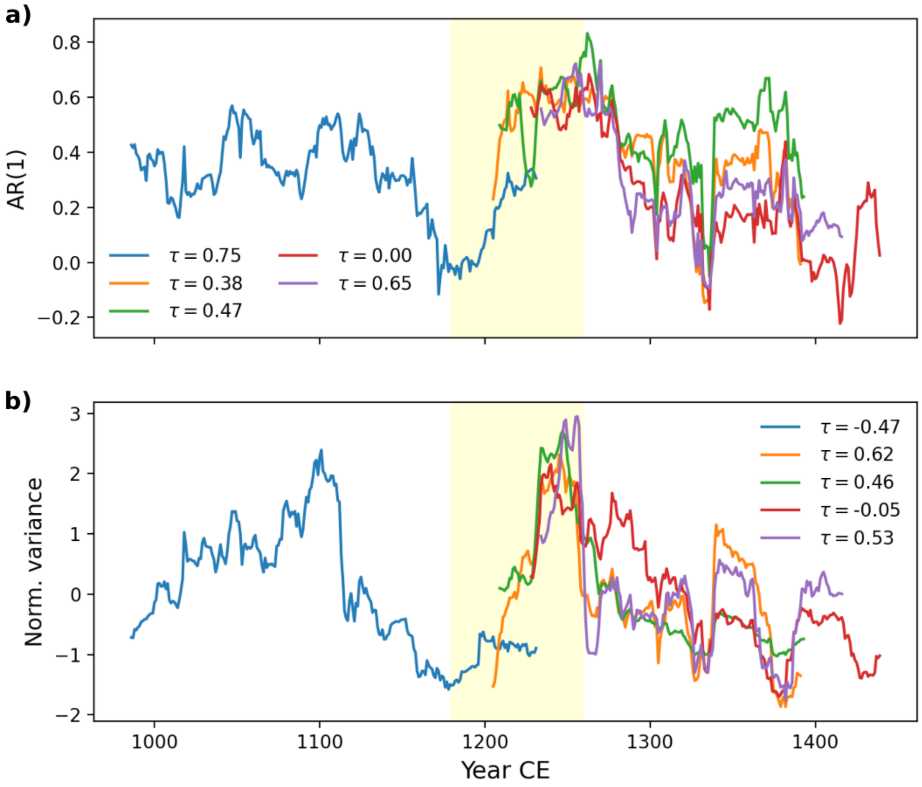

Supplementary Figure 6. Resilience indicators computed along increment widths measurements from individual shells before 1400. a) AR(1) and **b)** variance were computed along a 50-years sliding window over the detrended records. The age-related trend was removed by fitting a negative exponential curve after stabilising variance using a power-adaptive method. The first episode of loss of resilience is shaded in yellow.

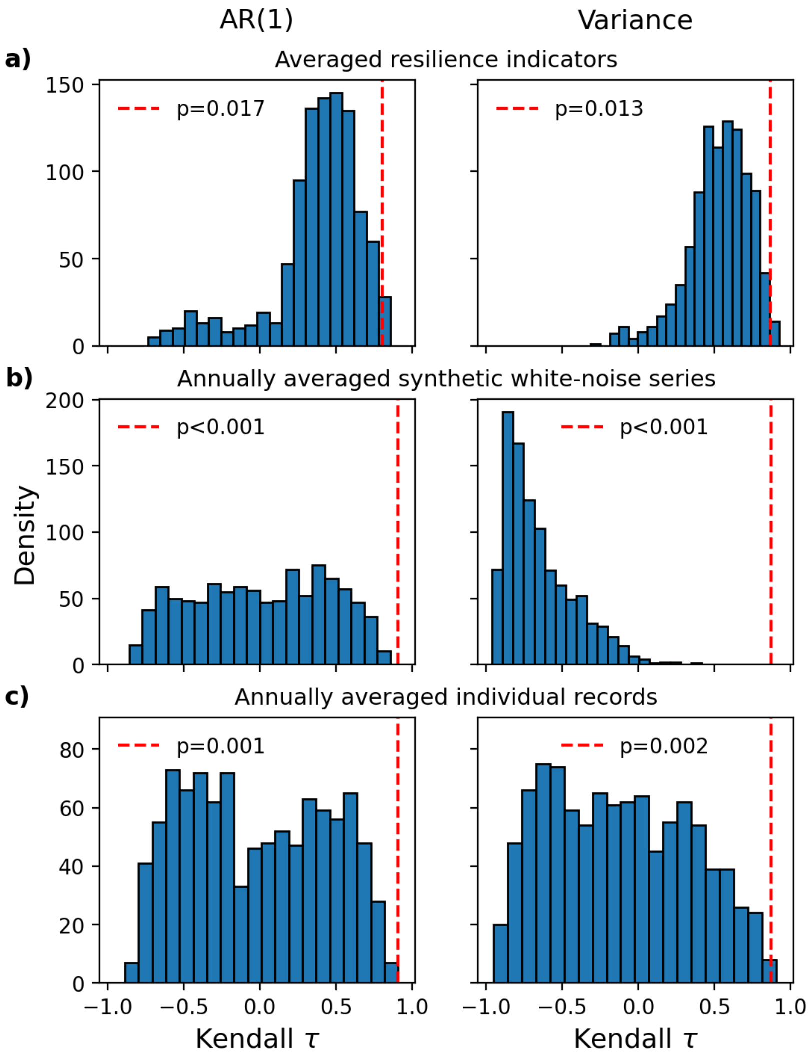

Supplementary Figure 7. Distribution of Kendall τ values expected from three ensembles, each of 1,000 realisations of a null model. Each realisation is a surrogate series built by introducing random shell-growth records at the same times as in the original chronology. The Kendall τ values correspond to the trends measured between 1200 and 1260. **a)** Each surrogate series is built by annually averaging the resilience indicators computed on the individual series. **b)** White-noise series are introduced instead of shell series to test the effect of the variable number of replicates through time. The white-noise series are averaged prior to computing the resilience metrics. **c)** The individual shell-growth records are averaged to obtain each surrogate series before computing the resilience metrics. The red dotted vertical line indicates the Kendall τ value measured on a series built following the process for each experiment but preserving the original order of the individual shell-growth records.

Supplementary References

1. Hald, M., Salomonsen, G. R., Husum, K. & Wilson, L. J. A 2000 year record of Atlantic Water temperature variability from the Malangen Fjord, northeastern North Atlantic. *The Holocene* **21**, 1049–1059 (2011).
2. Moffa-Sanchez, P., Hall, I. R., Thornalley, D. J. R., Barker, S. & Stewart, C. Changes in the strength of the Nordic Seas Overflows over the past 3000 years. *Quat. Sci. Rev.* **123**, 134–143 (2015).
3. Moffa-Sánchez, P. & Hall, I. R. North Atlantic variability and its links to European climate over the last 3000 years. *Nat. Commun.* **8**, 1726 (2017).
4. Good, S. A., Embury, O., Bulgin, C. E. & Mittaz, J. ESA Sea Surface Temperature Climate Change Initiative (SST_cci): Level 4 Analysis Climate Data Record, version 2.0. http://dx.doi.org/10.5285/aced40d7cb964f23a0fd3e85772f2d48 (2019).
5. Amante, C. & Eakins, B. W. ETOPO1 1 Arc-Minute Global Relief Model: Procedures, Data Sources and Analysis. http://dx.doi.org/10.7289/V5C8276M (2009).
6. Massé, G. *et al.* Abrupt climate changes for Iceland during the last millennium: Evidence from high resolution sea ice reconstructions. *Earth Planet. Sci. Lett.* **269**, 565–569 (2008).
7. Sejrup, H. P., Haflidason, H. & Andrews, J. T. A Holocene North Atlantic SST record and regional climate variability. *Quat. Sci. Rev.* **30**, 3181–3195 (2011).
8. Andersson, C., Risebrobakken, B., Jansen, E. & Dahl, S. O. Late Holocene surface ocean conditions of the Norwegian Sea (Vøring Plateau). *Paleoceanography* **18**, (2003).
9. Berner, K. S., Koç, N., Godtliebsen, F. & Divine, D. Holocene climate variability of the Norwegian Atlantic Current during high and low solar insolation forcing. *Paleoceanography* **26**, (2011).

10. Moffa-Sánchez, P., Born, A., Hall, I. R., Thornalley, D. J. R. & Barker, S. Solar forcing of North Atlantic surface temperature and salinity over the past millennium. *Nat. Geosci.* **7**, 275–278 (2014).

11. Jiang, H. *et al.* Solar forcing of Holocene summer sea-surface temperatures in the northern North Atlantic. *Geology* **43**, 203–206 (2015).

12. Lapointe, F. *et al.* Annually resolved Atlantic sea surface temperature variability over the past 2,900 y. *Proc. Natl. Acad. Sci.* **117**, 27171–27178 (2020).

13. Copard, K. *et al.* Late Holocene intermediate water variability in the northeastern Atlantic as recorded by deep-sea corals. *Earth Planet. Sci. Lett.* **313–314**, 34–44 (2012).

14. Tegzes, A. D., Jansen, E., Lorentzen, T. & Telford, R. J. Northward oceanic heat transport in the main branch of the Norwegian Atlantic Current over the late Holocene. *The Holocene* **27**, 1034–1044 (2017).

15. Reynolds, D. J. *et al.* Annually resolved North Atlantic marine climate over the last millennium. *Nat. Commun.* **7**, (2016).

16. Reynolds, D. J. *et al.* Biological and Climate Controls on North Atlantic Marine Carbon Dynamics Over the Last Millennium: Insights From an Absolutely Dated Shell-Based Record From the North Icelandic Shelf: δ13C dynamics of the North Atlantic. *Glob. Biogeochem. Cycles* **31**, 1718–1735 (2017).

17. Butler, P. G., Wanamaker, A. D., Scourse, J. D., Richardson, C. A. & Reynolds, D. J. Variability of marine climate on the North Icelandic Shelf in a 1357-year proxy archive based on growth increments in the bivalve Arctica islandica. *Palaeogeogr. Palaeoclimatol. Palaeoecol.* **373**, 141–151 (2013).

18. Cook, E. R. & Peters, K. Calculating unbiased tree-ring indices for the study of climatic and environmental change. *The Holocene* **7**, 361–370 (1997).
